# RASopathies and Cardiac Complications: Insights into Mechanisms, Diagnosis, and Innovative Treatments

**DOI:** 10.2174/011573403X341624250324164700

**Published:** 2025-03-28

**Authors:** Keshav Garg, Shubam Trehan, Fremita Fredrick, Ankur Singla, Kanishk Aggarwal, Aachal Gupta, Rohit Jain

**Affiliations:** 1 Government Medical College, Chandigarh, India;; 2 Dayanand Medical College & Hospital, Ludhiana, India;; 3 Avalon University School of Medicine, Willemstad, Curacao;; 4 PGY1 Resident Internal Medicine, Northwest Health-Porter, Valparaiso, IN 46383, USA;; 5 Amity Regional High School, Woodbridge, CT 06525, USA;; 6 Department of Internal Medicine, Penn State Milton S. Hershey Medical Center, Hershey, PA 17033, USA

**Keywords:** RASopathies, hypertrophic cardiomyopathy, cardiac hypertrophy, RAS gene mutations, RAS/MAPK pathway, MEK inhibitors, congenital heart disease, cardiovascular genetics

## Abstract

RAS proteins are critical in cellular signal transduction, influencing cell proliferation, differentiation, and survival. While extensively studied for their role in cancer, RAS gene mutations also contribute significantly to cardiovascular diseases, such as hypertrophic cardiomyopathy, pulmonary valve stenosis, and atrial septal defects. Despite their similar primary structures, RAS proteins exhibit distinct functions in cardiac biology: H-RAS regulates cardiomyocyte size, K-RAS governs proliferation, and N-RAS, less associated with cardiac defects, is understudied in cardiac cells. Congenital RAS mutations, collectively known as RASopathies, include syndromes, like Noonan syndrome and cardio-facio-cutaneous syndrome, which often lead to severe cardiac complications, including heart failure. Genetic testing and imaging advances have improved the diagnosis and management of these conditions. Recent research has shown promise with MEK inhibitors and other targeted therapies, offering potential improvements in managing RAS-related cardiac conditions. This review explores the role of the RAS subfamily in heart disease, highlighting key concepts and potential therapeutic targets. PubMed database was searched using keywords, such as RASopathies, RAS gene mutations, cardiac hypertrophy, cardiovascular disease, RAS/MAPK pathway, congenital heart disease, and more. Relevant literature up to June 2024 was examined and summarized, consisting of data from various clinical trials, meta-analyses, retrospective/prospective cohort studies, and current guidelines.

## 1 INTRODUCTION

Signal transducers play an essential role in cellular communication, translating extracellular signals into intracellular actions that regulate various cellular processes. Among these transducers, the RAS (Rat sarcoma virus) family of genes is particularly significant due to its involvement in cell proliferation, differentiation, and survival [1]. The RAS gene family, including Kirsten rat sarcoma virus (K-RAS), Harvey rat sarcoma virus (H-RAS), and neuroblastoma rat sarcoma virus (N-RAS), encodes small guanosine triphosphate (GTP)-binding proteins that function as molecular switches, controlling signal transduction pathways. While mutations in RAS genes are well-documented in cancer pathogenesis, their roles in cardiovascular diseases are increasingly recognized [2, 3].

RAS proteins and their associated pathways significantly influence the heart's physiological and pathological development, playing crucial roles in shaping cardiac phenotypes [4]. Mutations in genes encoding RAS pathways are linked to hypertrophic cardiomyopathy (HCM), pulmonary valve stenosis (PVS), and atrial septal defects (ASD). RASopathies include conditions, like Noonan syndrome (NS), cardio-facio-cutaneous (CFC) syndrome, neurofibromatosis type 1 (NF1), Costello syndrome (CS), and Legius syndrome (LS), which arise from mutations in the RAS/ mitogen-activated protein kinase (MAPK) signaling pathway, leading to diverse clinical manifestations, prominently featuring cardiovascular complications. While individual RASopathies are rare, collectively, they represent the most common genetic condition, affecting 1 in every 1000-2500 live births [5]. Activating somatic RAS gene mutations exist in about 30% of human cancers [6]. Notably, RASopathies are diagnosed in approximately 18% of childhood HCM cases, and among infants younger than one year, they account for around 42% of these cases [7]. Studies indicate that cardiac defects are present in 80.3% of congenital RASopathy cases, with an overall crude mortality rate of 0.29 per 100 patient-years [8]. The activation or dysregulation of the Ras/MAPK pathway results in common clinical features of RASopathies, affecting the cardiovascular, lymphatic, musculoskeletal, cutaneous, and central nervous systems. Heart defects associated with RASopathies include congenital heart disease (CHD), HCM, and dilated cardiomyopathy (DCM), and the prognosis is heavily influenced by the severity of cardiac involvement and other comorbidities [9]. Patients with HCM in RASopathies may have a variable prognosis, with infants diagnosed with severe HCM within the first six months of life often having a poor prognosis and a 2-year survival rate of approximately 30% [10]. While a study found an overall survival rate of 94.3% in RASopathy patients, severe cardiac issues, like HCM, significantly impact mortality [11]. The average age of survival varies, with many patients living into adulthood but facing higher risks of cardiac complications and malignancies. Genetic testing and imaging technology advances have improved diagnosis and monitoring, management, and outcomes of the disease [12].

Despite RAS proteins' critical roles in cardiac function, significant gaps remain in our knowledge regarding the mechanisms by which these proteins contribute to cardiovascular pathologies. Current treatments for heart failure (HF) and other cardiovascular diseases often fail to address the underlying molecular abnormalities, highlighting the need for novel therapeutic approaches [4].

Recent advancements in understanding RASopathies have driven research into innovative treatments, mainly targeting the RAS pathway. Mitogen-activated protein kinase kinase (MEK) inhibitors, such as trametinib, cobimetinib, vemurafenib, and binimetinib, show promise in blocking the RAS/MAPK pathway, alleviating cardiac symptoms, like hypertrophy, and improving outcomes in both animal models and human cases [12]. Combination therapy with trametinib and everolimus/sirolimus has significantly benefited infants with HCM [13]. CH5126766, a dual rapidly accelerated fibrosarcoma (RAF)/MEK inhibitor, effectively reduces tumor size in RAS-mutated models [14]. In ischemic heart conditions, farnesyl protein transferase inhibitors and high-dose lovastatin help preserve left ventricular function [15, 16]. Telmisartan can reduce fibrosis and atrial fibrillation (AF) by inhibiting the RAS-extracellular signal-regulated kinase (ERK) pathway and enhancing PI3K-Akt-eNOS signaling [3, 17]. Other approaches include statins, farnesyl transferase inhibitors (FTIs), and small molecules, like cyclosporine and rapamycin, which target the RAS signaling pathway to treat cardiac hypertrophy and HF [3, 18, 19].

This paper aimed to provide a comprehensive overview of the RAS subfamily and its signaling pathways in cardiac disorders, mainly focusing on cardiac hypertrophy and HF. The goal was to identify potential therapeutic targets by exploring associated genetic abnormalities.

## 2 PATHOPHYSIOLOGY

Signal transducers constitute one of the categories of proto-oncogenes, responsible for carrying the signal generated due to the interaction of growth factor and growth factor receptor with the nucleus. The RAS gene is a family of signal transducer molecules prone to undergo point mutation in rare cases, causing many types of cancer, including melanoma, lymphoma, and various other hemato-oncologic carcinomas [1, 2]. Their role in cardiac physiology and various cardiovascular pathologies is also being recognized increasingly. The three primary RAS genes found in humans, including K-RAS, H-RAS, and N-RAS, encode for four isoforms within the cells, *i.e.,* H-RAS, N-RAS, K-RAS4A, and K-RAS4B, with the latter two resulting from alternate splicing of the K-RAS gene [20]. K-RAS is associated with cardiac hyperplasia, while H-RAS is associated with cardiac hypertrophy, and N-RAS has minor functional importance in the heart, although its active mutations cause NS [21-23].

### 2.1 RAS Pathway

The RAS signaling network connects extracellular receptors and various intracellular pathways. The extracellular receptors relay the signals to the RAS network, which activates the internal pathways. This results in the activation of several downstream effectors, causing various metabolic effects. G-protein coupled receptors (GPCRs) and receptor tyrosine kinases (RTKs) are examples of the various classes of extracellular receptors related to the RAS pathway [12].

The RTKs, when activated by different hormones, cytokines, and polypeptide growth factors, undergo phosphorylation and activate various downstream pathways. These phosphor-tyrosines bind to adaptor proteins, such as sarcoma (src) homology and collagen (SHC)/growth factor receptor-bound 2 (GRB2) complex, which then bind to the most vital guanine nucleotide exchange factor (GEF) of RAS, the cytosolic protein son of sevenless (SOS), and thus, activate RAS [24]. Other adaptor proteins include src homology (SH2) domain-containing protein tyrosine phosphatase (SHP2) and GRB2- associated-binding protein (GAB). Shp2s interact with GAB and RTK to induce RAS signaling.

With RAS's inefficient spontaneous activity, GEFs, like SOS and exchange protein directly activated by cyclic adenosine monophosphate (cAMP), activate RAS genes by exchanging guanosine diphosphate (GDP) for GTP, leading to downstream signaling and cellular proliferation. GTPase-activating proteins (GAP), such as neurofibromin 1 and carabin, hydrolyze GTP to GDP, leading to the downregulation of RAS [24, 25].

GPCRs activate the RAS pathway mainly *via* two major pathways: the phosphatidylinositol signaling pathway, which is Gq receptor coupled, and the cAMP signaling pathway, which is Gs receptor coupled. Catecholamines, like norepinephrine (NE), activate β-adrenergic receptors (a type of Gs receptor), leading to the activation of adenylate cyclase, which produces cAMP. cAMP further activates protein kinase A (PKA) that, *via* an interlink between various proteins [like L‐type calcium channel (LTCC), ryanodine receptor (RyR), phospholamban (PLN), and sarco-endoplasmic reticulum Ca++ adenosine triphosphate (ATP)‐ase (SERCA)], facilitates calcium (Ca2+) cycling and increases Ca2+ in the cytoplasm. cAMP also directly activates the Epac, one of the GEFs [26]. Meanwhile, after being stimulated by either angiotensin II (AgII) or NE, the Gq receptors cause the activation of phospholipase C (PLC). PLC causes the hydrolyzation of phosphatidylinositol‐4,5‐bisphosphate to inositol‐1,4,5‐trisphosphate and diacyl‐glycerol, which eventually results in an increase in Ca2+ in the cytoplasm, leading to downstream effects similar to the above cAMP pathway. Gq is also capable of activating RAS directly [3].

**Fig. (1):**
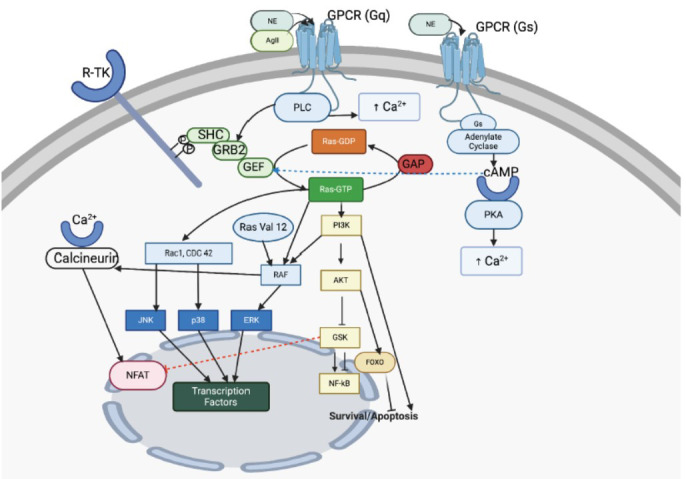
Upstream and downstream processing of the RAS pathway; **Upstream processing -** The RAS signaling pathway is activated by extracellular receptors, such as receptor tyrosine kinases (RTKs) and G-protein-coupled receptors (GPCRs). When RTKs bind to ligands, they phosphorylate and activate adaptor proteins (SHC/GRB2, Src homology, and collagen/growth factor receptor-bound 2), which recruit and activate the guanine nucleotide exchange factor (GEF). This causes the exchange of GDP for GTP on RAS, converting it to its active form, RAS-GTP. GPCRs, when activated by ligands, like norepinephrine (NE) and angiotensin II (AgII), activate either the phosphatidylinositol (Gq), *via* phospholipase C (PLC) or cAMP (cyclic adenosine monophosphate) (Gs) or protein kinase A (PKA) signaling pathways, increasing intracellular Ca2+ levels and further activating RAS. GTPase activating proteins (GAP), by hydrolyzing GTP to GDP, downregulate RAS. **Downstream processing -** Active RAS-GTP triggers several downstream effectors, including RAF (rapidly accelerated fibrosarcoma), PI3K (phosphoinositide 3-kinase), RAC1 (Ras-related C3 botulinum toxin substrate 1), and CDC 42 (cell division control protein 42). This activation cascades through multiple pathways, defined as follows: A) MAP kinase pathways: RAF activates ERK (extracellular signal-regulated protein kinase), JNK (c-Jun NH2-terminal kinase), and p38, leading to transcription factor activation [*e.g.*, JUN, myocyte enhancer factor-2 (MEF2), GATA binding protein-4 (GATA-4)]. B. PI3K/AKT pathway: PI3K activates AKT (protein kinase B), which activates nuclear factor kappa B (NF-κB), inhibits nuclear factor of activated T-cells (NFAT) and glycogen synthase kinase (GSK), and also influences Forkhead box transcription factors of class O (FOXO). C) Calcineurin pathway: Increased Ca2+ levels activate calcineurin, which promotes NFAT nuclear translocation and transcriptional activation. These pathways collectively regulate cardiac hypertrophy, survival, and apoptosis. BioRender.com.

The increased cytosolic Ca2+, due to either pathway, can bind to calcineurin, and once activated, calcineurin causes activation and nuclear translocation of the nuclear factor of activated T cells (NFAT‐3). Ca2+/calmodulin signaling pathway is also activated by the increased cytoplasmic Ca2+ concentration [3]. Once activated, RAS causes activation of various MAPK pathways *via* RAF, such as ERK, Jun NH2-terminal kinase (JNK), p38, and some non-kinase pathways, such as Ca2+/calcineurin pathway, and the protein kinase B (Akt)/mammalian target-of-rapamycin (mTOR) pathway. While the ERK pathway causes physiological cardiac hypertrophy, the JNK and p38, both known as stress-activated protein kinases (SAPK) pathways, can be activated by RAS *via* RAS-related C3 botulinum toxin substrate 1 (RAC1) or cell division control protein 42 (CDC 42), along with the Ca2+/calcineurin pathway, leading to pathological hypertrophy. All these pathways do so by causing activation of transcriptional factors, like JUN, myocyte enhancer factor-2 (MEF2), and GATA binding protein-4 (GATA4) *via* calcineurin or NFAT [24, 27]. Akt activated by the RAS pathway *via* phosphoinositide 3‐kinase (PI3K) has a protective effect on the cardiac muscle *via* activation of nuclear factor kappa B (NF-κB) and inhibition of NFAT. Akt can also lead to inhibition of glycogen synthase kinase (GSK), which is also cardioprotective and causes activation of forkhead box transcription factors of class O (FOXO), which reduces apoptosis and is thus bad for cardiac health [28]. The PI3K/Akt/endothelial nitric oxide synthase (eNOS) pathway, which involves the release of nitric oxide after stimulation from Akt, is also responsible for maintaining cardiac contractility and vascular hemostasis (Fig. **1**) [29].

### 2.2 The Effects of RAS Pathway on the Cardiovascular System

RASopathies are a group of inherited genetic syndromes, including mutations of RAS-MAPK nucleotides: tyrosine-protein phosphatase non-receptor type 11 (PTPN11), SOS1, RAF1, BRAF, KRAS, HRAS, and NRAS [30, 31]. Germline mutations in a person’s cells display distinct characteristics as opposed to somatic RAS mutations, which are mostly associated with oncogenesis [6]. These mutations affect normal cellular proliferation, differentiation, and apoptosis by hyperactivating downstream signaling pathways, even though they are not usually in the RAS genes themselves. About 50% of cases of NS, the most prevalent RASopathy, have mutations in PTPN11 [32]. Variants, such as p.N308D, increase SHP2 activity, thereby increasing downstream RAS-MAPK signaling and causing HCM [33]. Similarly, mutations of RAF1, including p.S257L, have been reported in over 75% of the cases of NS with HCM, hence establishing the critical role the gene plays in cardiac remodeling and development [34, 35]. Mutations of HRAS, such as p.G12S and p.G12C, are pathogenic risk factors in the development of CS. Other anomalies that have been associated with these mutations include valvular defects and abnormal heart rhythms [36, 37]. Moreover, HRAS mutations have been shown to impact transcriptional regulation of atrial natriuretic factor and the organization of sarcomeres, resulting in cardiomyocyte augmentation and subsequent remodeling [22].

The activation of RAS is a potent mechanism responsible for inducing cardiac hypertrophy, which ultimately leads to HF and myocardial fibrosis, as per some animal studies. According to a study by Haq *et al*., the kinase pathways, including SAPK and p38, and the Akt pathway, are highly active in HF [38]. In cardiac hypertrophy, the Ca2+/calcineurin pathway has been found to be highly active, and it has also been found that the oncogenic variant of HRAS causes activation of the Ca2+/calcineurin pathway [3]. The most commonly reported mutation is the GTP-binding protein or GAP mutation, making it difficult to shut off the massive RAS signaling, leading to the overgrowth of particular bodily cells and tissues, including cardiac hypertrophy and HF [3]. It has also been found that RAS-ERK and the PI3/Akt pathways are activated by hypertension, which eventually results in remodeling of the heart and cardiac arrhythmias, like atrial arrhythmias. Expression of the ERK pathway has also been found to be high in patients having AF [39]. HCM, PVs, and ASD are the most common cardiac diseases found in patients having mutations in the genes encoding the RAS pathways [40]. These syndromes are known as RASopathies, which include conditions, like NS, CS, and CFC [12]. RAS valine (val)-12, an oncogenic mutant of HRAS, causes activation of the Ca2+/calcineurin pathway *via* activation of RAF and leads to pathological cardiac hypertrophy, similar to HCM [41]. The RAS pathway, *via* the hyperplasia suppressor gene and the PI3K/Akt/eNOS pathway, has also been found to affect atherosclerosis-related vascular smooth muscle remodeling [29, 42].

Emerging research has identified the importance of non-coding regulatory mutations in modulating RAS pathway dynamics [43]. Variants of genes, like SOS1 and RAS guanine nucleotide-releasing protein1 (GRP1), influence phenotypic variability, particularly in congenital heart defects, like ASD and valve abnormalities [44]. Murine models carrying KRAS V14I mutations have recapitulated NS phenotypes, including unique patterns of ventricular fibrosis and hypertrophy, providing valuable insights into the structural cardiac anomalies observed in RASopathies [21]. These findings highlight the diverse genetic landscape of RASopathies, necessitating a system biology approach to fully understand their molecular and phenotypic complexity.

### 2.3 Advances and Limitations of Therapeutics for RASopathies

Although the therapeutic landscape for RASopathies is moving into positive territories, significant issues still predominate. Trametnib and selumetinib, which are MEK inhibitors, seem to have a beneficial effect on the management of HCM and cardiomyopathy associated with RASopathies *via* the dampening of overactive ERK pathways [45]. Notwithstanding, their side effects include immune suppression, gastrointestinal disturbances, and growth retardation, which hinder their use in the pediatric age group [46]. Recently, preclinical studies have tested the inhibition of Shp2 to be more specific at the PTPN11 mutation hyperactivity site, but its effectiveness in the treatment of cardiac phenotypes has not yet been fully established [47].

Innovative technologies, like clustered regularly interspaced short palindromic repeats (CRISPR)-associated protein 9 (Cas9) gene editing, exhibit promise to be able to reverse pathogenic variants, especially some missense mutations [48]. There are other experiments in progress, for instance, the National Cancer Institute’s advancing RAS/RASopathy therapies, whose goal is to find suitable treatment and prevention options for non-NF1 RASopathies and RAS-mutated tumors [49]. More recent research has indicated RASopathy mutations to have a wide range of treatment efficacy, warranting the use of personalized medicine approaches to target their specific genetic and phenotypic characteristics [50]. Even further, progress made in imaging biomarkers and *in silico* modeling adds the capability to customize treatment plans [51]. Despite these advancements, the lack of therapies addressing tissue-specific consequences and compensatory mechanisms within the RAS pathway underscores the need for continued research and innovation.

## 3 DISCUSSION

### 3.1 RASopathies

NS is a relatively common autosomal dominant genetic disorder with an estimated prevalence of 1 in 1,000 to 1 in 2,500 live births, though rare autosomal recessive forms exist [52, 53]. It is caused by gain-of-function mutations in genes of the RAS/MAPK signaling pathway, which is crucial for regulating cell proliferation, differentiation, and survival. Approximately 50% of cases result from mutations in PTPN11, which encodes the tyrosine phosphatase SHP2, while other implicated genes include SOS1, RAF1, KRAS, NRAS, RAS-related (R-RAS), and leucine zipper-like transcription regulator 1 (LZTR1) [32]. These mutations disrupt normal regulatory mechanisms, leading to hyperactivation of RAS/MAPK signaling and the associated phenotypic manifestations. NS is clinically characterized by distinct facial dysmorphia, congenital heart defects (*e.g.*, pulmonary valve stenosis, hypertrophic cardiomyopathy), proportional short stature, and skeletal abnormalities, such as decreased bone mineral density. Neurocognitive deficits, delayed puberty, cryptorchidism, and increased cancer risk, including juvenile myelomonocytic leukemia (JMML) and rhabdomyosarcoma, are also notable features [54]. Genotype-phenotype correlations further refine the clinical spectrum; for example, PTPN11 mutations are strongly associated with hypertrophic cardiomyopathy, while SOS1 mutations often present with ectodermal abnormalities [52].

NS with multiple lentigines (NSML) is a rare autosomal-dominant condition caused by specific mutations in PTPN11, predominantly affecting its phosphatase domain. Unlike NS, these mutations lead to a decrease in phosphatase activity, but they share overlapping clinical features, such as craniofacial dysmorphia and HCM [55]. Patients with NSML face increased risks of malignancies, such as neuroblastoma and acute myelogenous leukemia. NSML is further distinguished by sensorineural hearing loss, multiple lentigines (skin pigmentation), and ocular hypertelorism. HCM and electrocardiographic abnormalities are more prominent in NSML compared to NS, while PVS is less common [52].

CS is a rare autosomal dominant disorder caused by *de novo* pathogenic variants in HRAS, primarily the G12S mutation. It is considered a cancer predisposition syndrome, as it shares HRAS variants with malignancies. Patients with CS have an elevated risk of rhabdomyosarcoma, neuroblastoma, and early-onset bladder carcinoma [56]. Clinically, CS presents with distinct craniofacial features (macrocephaly, prominent forehead), cutaneous abnormalities (papillomas), hypotonia, joint laxity, and cognitive deficits [57-62]. Congenital heart defects, particularly HCM, are common. Perinatal complications, including polyhydramnios, prematurity, and failure to thrive, are frequently reported [59, 60].

CFC is a rare autosomal-dominant disorder caused by mutations in BRAF (75% of cases), MAP2K1 (MEK1), MAP2K2 (MEK2), or less frequently, KRAS [63-65]. Pathogenic variants in BRAF and MEK1/2 increase MEK/ERK phosphorylation, amplifying RAS/MAPK signaling and driving the disorder's pathogenesis [52]. This syndrome overlaps with NS in some features, but has unique dermatological characteristics, including thin, brittle hair; sparse eyebrows; hyperkeratotic skin; and hemangiomas. Neurological symptoms, such as cognitive impairment, epilepsy, and speech delays, are more severe in CFC compared to NS [63].

LS is a rare autosomal dominant RASopathy caused by mutations in sprouty-related EVH1 domain containing 1 (SPRED1), which impairs the recruitment of NF1 to the plasma membrane for its RAS GAP function [64, 65]. SPRED1 deficiency leads to enhanced RAS/MAPK signaling, as shown in SPRED1 knockout models, which exhibit behavioral and craniofacial abnormalities. MEK inhibition rescues some LS-associated phenotypes, particularly social deficits, though other cognitive issues remain unresolved [52]. LS often mimics NF1, presenting with café-au-lait macules, but lacking neurofibromas. Other features include mild facial dysmorphia, musculoskeletal abnormalities (*e.g.*, pectus deformities), learning disabilities, and attention deficit hyperactivity disorder (ADHD) [66, 67].

### 3.2 Cardiovascular Presentation and Symptoms

RAS proteins are crucial in cardiac pathology, impacting conditions, like coronary artery disease, hypertrophy, and HF. Genome-wide association studies have highlighted the significance of MRAS variants in coronary artery disease, particularly among Han Chinese populations, due to their unique genetic makeup that predisposes them to certain conditions [68]. These variants are associated with dyslipidemia, including obesity, elevated cholesterol, and triglyceride levels, which are established risk factors for coronary artery disease. Dysregulation of R-RAS has also been implicated in vascular instability observed in hyperglycemia and tumor angiogenesis. Additionally, R-RAS2 plays a critical role in regulating platelet function and thrombus stability, which is crucial for vascular homeostasis and disease progression [69]. Patients presenting with HCM progress through several stages as follows: stage I manifests with mild symptoms, like decreased exercise tolerance and left ventricular (LV) hypertrophy, while stage II involves adverse myocardial remodeling with LV dysfunction, diastolic dysfunction, and potential arrhythmias. Stage III marks end-stage disease with extensive fibrosis, LV dilatation, and high mortality, typically presenting between ages 20 and 50, but variably across different age groups [70].

In congenital RASopathies, a significant proportion of patients experience CHD, with PVS being the most prevalent and conditions, like tetralogy of Fallot and patent ductus arteriosus, being rare. Additional cardiac anomalies include DCM, ASD, or ventricular septal defects, atrioventricular canal defects, and various left-sided defects, such as mitral valve stenosis, aortic valve stenosis, and aortic coarctation. HCM affects approximately 20% of individuals with RASopathies, often diagnosed early in infancy [12, 71]. RASopathy-related HCM is associated with a notable prevalence of sudden cardiac death (SCD) at 4%, translating to an annual incidence rate of 0.86 per 100 person-years. Retrospective studies have identified that 60.9% of patients with RASopathy-related HCM harbor mutations in the RAS-MAPK pathway. Among these patients, 16.6% have died from various causes, including congestive HF (28.6%), SCD (10.7%), non-cardiac causes (21.4%), and unknown etiologies (39.3%). Additionally, 6.5% have experienced SCD-equivalent events, comprising SCD (27.3%), aborted cardiac arrest (45.5%), appropriate implantable cardioverter-defibrillator shock (9.1%), and sustained ventricular tachycardia (18.2%) [72].

### 3.3 Diagnosis

Imaging and histology are crucial for detecting myocardial abnormalities, like hypertrophy of myocardial fibers and interstitial fibrosis, especially in DCM and HCM. At the same time, baseline cardiac evaluations, including electrocardiograms and echocardiograms, are recommended at diagnosis, with follow-up protocols tailored to CHD or HCM. Imaging techniques and genetic analysis also assist in diagnosing related conditions, like neurofibromatosis, which presents with neurofibromas that can affect the heart and major vessels, leading to various complications [9, 12]. A study comparing RASopathy-related HCM to primary HCM revealed significant echocardiographic differences. Patients with RASopathy-related HCM had higher median LV posterior wall diameter z-scores (2.9 *vs*. 2.5; *P* = 0.029) and greater baseline LV outflow tract gradients (20 mm Hg *vs*. 13.5 mm Hg; *P* = 0.002) [73]. Combining clinical evaluations with genetic testing is crucial for determining specific causes and associated risks of congenital RASopathies. Molecular diagnosis through gene resequencing panels is widely accessible and vital for identifying RASopathies. For instance, over 50% of patients with NS have pathogenic variants in the protein tyrosine phosphatase non-receptor type 11 gene, leading to hyperactivation of the RAS/MAPK pathway, which can be detected through genetic testing. However, despite the availability of these tests, they have yet to be universally applied to patients with CHD, although they are essential for accurate diagnosis and prognostication (Table **1**) [63].

### 3.4 Treatment

Recent advancements in RASopathies have propelled research into novel treatment strategies, mainly focusing on inhibitors of the RAS pathway. The management currently revolves around interventions, like percutaneous balloon valvuloplasty and surgical valvotomy, for severe cases [63]. Recent advancements in RASopathies have led to promising research into novel treatment strategies, particularly MEK inhibitors. These inhibitors, such as trametinib, cobimetinib, vemurafenib, and binimetinib, show potential in blocking the RAS/MAPK pathway, thereby alleviating cardiac symptoms, like hypertrophy, and improving fractional shortening in both animal models and selected human cases [12]. In 2020, the MEK inhibitor selumetinib received approval for use in children aged two years and older diagnosed with neurofibromatosis type 1, which hyperactivates the RAS-MAPK pathway, particularly those with symptomatic and inoperable plexiform neurofibromas. This approval was based on clinical trials that showed a 66% overall response rate, with 82% of those responding maintaining their response for over 12 months [74]. Combination therapy with trametinib and everolimus/sirolimus has demonstrated significant clinical benefits in HCM treatment, including reduced mortality, surgery, and transplantation needs, particularly in infants under six months. Techniques, like ATP analog-sensitive ERK and immobilized metal affinity chromatography, provide insights into RAS activity, aiding the evaluation of MEK inhibitors' effectiveness [75]. Another investigation by Nakagawa *et al*. [14] assessed CH5126766, a dual RAF/MEK protein inhibitor, in managing RAS-mutated rhabdomyosarcoma (RMS). It effectively suppressed MEK activity and RAF-mediated MEK phosphorylation, resulting in robust inhibition of ERK signaling compared to conventional MEK inhibitors. Notably, CH5126766 exhibited selective growth inhibition of RAS-mutated RMS cell lines at lower concentrations relative to non-mutated counterparts. *In vivo* experiments using RMS xenograft models confirmed these results, showing a significant reduction in tumor size without inducing apoptosis *in vitro*, being consistent with findings from other studies investigating inhibitors of the MAPK pathway [14].

In RAS-related ischemic heart conditions, a study found that pre-treatment with farnesyl protein transferase-III and high-dose lovastatin showed protective effects by preserving left ventricular function, although efficacy diminished during reperfusion [15, 16]. Hypertension, a significant risk factor for AF, induces structural changes and conduction abnormalities *via* the renin-angiotensin-aldosterone system. Studies have shown that telmisartan, compared to valsartan, can downregulate these effects by inhibiting the RAS-ERK pathway and enhancing PI3K-Akt-eNOS signaling, thereby reducing myocyte size, fibrosis, and AF incidence [3, 75]. Statins inhibit farnesyl synthesis through HMG-CoA reductase, and they have demonstrated efficacy in trials involving animal models, especially at high doses. However, clinical trials involving human RASopathy patients using statins have shown varied outcomes, indicating modest benefits, whereas FTIs, initially explored in cancer therapies, have been reported to block farnesyl pyrophosphate synthase, yielding positive results in cardiac remodeling models [3]. Carabin, a Ras-GAP protein, regulates calcineurin and RAS signaling, and can be a potential candidate for treating cardiac hypertrophy and HF [18]. Raf kinase inhibitor protein enhances beneficial β-adrenergic pathway activation while decreasing adverse remodeling effects [19]. Medications, like cyclosporine and rapamycin, also target RAS pathways, with cyclosporine inhibiting calcineurin to mitigate cardiac hypertrophy, especially with HF risks. Rapamycin inhibited JNK and Akt/mTOR pathways, reversing hypertrophy in genetic models (Table **2**) [3].

## 4 FUTURE DIRECTIONS

Improving the management of RASopathy-associated cardiac complications requires overcoming current therapeutic limitations and pursuing innovative approaches. While treatments, such as MEK and mTOR inhibitors, show promise, their utility is often restricted by side effects, like growth suppression and immune dysfunction, particularly in children [12, 46]. Refining these therapies and developing predictive biomarkers to enable personalized medicine are essential steps forward [50, 51]. Gene-editing technologies, such as CRISPR-Cas9, offer a groundbreaking potential to address pathogenic mutations, though challenges, like achieving tissue-specific targeting and avoiding unintended effects, remain [48, 49]. Advances in precision medicine, supported by genetic profiling and computational modeling, could enable tailored treatment strategies for distinct mutations and clinical presentations [50, 51]. Furthermore, identifying new molecular targets, including Shp2 inhibitors and other pathway-specific drugs, alongside advancements in imaging techniques and biomarkers, may improve early detection and treatment evaluation [12, 47, 51, 63]. Research on non-coding regulatory mutations could also reveal novel mechanisms behind congenital heart defects, opening additional therapeutic pathways [43, 44]. Collaborative multicenter studies are crucial for gathering longitudinal data, enhancing therapeutic approaches, and deepening our understanding of RASopathies [47, 63]. These integrated efforts aim to reduce the impact of RASopathy-related cardiac conditions and foster better outcomes through innovation, precision, and teamwork.

## CONCLUSION

RAS proteins’ involvement in cardiovascular diseases, particularly in conditions, such as HCM, PVS, and ASD, underscores their significance in cardiac health and disease. RAS gene dysregulation, in conditions collectively known as RASopathies, highlights the importance of the Ras/MAPK pathway in developing cardiac abnormalities and other systemic manifestations. Accurate diagnosis of RASopathies relies on advanced imaging techniques and genetic analysis. Imaging and histology are crucial for detecting myocardial abnormalities, like hypertrophy of myocardial fibers and interstitial fibrosis, especially in DCM and HCM. Baseline cardiac evaluations are recommended at diagnosis, with follow-up protocols tailored to CHD or HCM. Genetic testing is essential for identifying specific mutations in the RAS/MAPK pathway, aiding in the prognosis and management of these conditions. The pathophysiology of RASopathies involves dysregulation of the Ras/MAPK pathway, leading to altered cell growth, differentiation, and survival. Mutations in the genes encoding components of this pathway cause hyperactivation, resulting in abnormal cardiac development and function. In HCM, for instance, excessive Ras signaling leads to myocardial hypertrophy, fibrosis, and diastolic dysfunction. In PVS and ASD, disrupted Ras signaling impairs normal valvular and septal formation, contributing to structural heart defects. Recent research on MEK inhibitors, such as trametinib and cobimetinib, has shown promise in alleviating cardiac symptoms associated with RASopathies. These inhibitors target the Ras/MAPK pathway, potentially mitigating cardiac hypertrophy and improving outcomes in both animal models and human patients. Furthermore, combination therapies, such as MEK inhibitors with mTOR inhibitors, have demonstrated significant benefits in managing HCM. Other therapeutic strategies, including statins, FTIs, and small molecules, like cyclosporine and rapamycin, also show potential in modulating the Ras pathway to treat cardiac hypertrophy and HF. These treatments aim to correct the underlying molecular defects, offering hope for improved management of RAS-related cardiac pathologies. As our understanding of the Ras signaling network and its impact on cardiac health continues to evolve, the development of targeted therapies holds promise for better outcomes for patients with RASopathies. Further research is essential to bridge the current knowledge gaps and refine therapeutic strategies, which can ultimately lead to more effective and personalized treatments for those affected by these complex genetic conditions.

## Figures and Tables

**Table 1:** Diagnostic modalities for RAS-related cardiovascular complications.

**Imaging and histology**	Detects myocardial abnormalities (*e.g.*, hypertrophy and interstitial fibrosis) in DCM and HCM.
**Baseline evaluations**	Electrocardiograms and echocardiograms are recommended at diagnosis.
**Echocardiographic differences**	Median LV posterior wall diameter z-scores and baseline LV outflow tract gradients are higher in RASopathy-related HCM *vs*. primary HCM.
**Genetic testing**	It is crucial for diagnosing congenital RASopathies. Gene sequencing panels are widely accessible.

**Note:** [12, 37, 38].

**Table 2:** Treatments for RAS-related cardiovascular complications.

**MEK inhibitors**	• MEK inhibitors, such as trametinib, cobimetinib, vemurafenib, and binimetinib, alleviate cardiac symptoms. • Selbutinib has been recommended for children aged 2+ witd neurofibromatosis type 1 and symptomatic, inoperable plexiform neurofibromas.
**Combination therapy (MEK inhibitors + mTOR inhibitors)**	• Trametinib with everolimus/sirolimus reduced mortality, surgery, and transplantation needs in HCM, especially in infants.
**CH5126766 (dual RAF/MEK inhibitor)**	• It effectively suppressed MEK activity and RAF-mediated MEK phosphorylation. • Robust ERK signaling was inhibited in RAS-mutated RMS cell lines, causing significant tumor size reduction *in vivo.*
**Farnesyl protein transferase-III and lovastatin**	• Protective effects in RAS-related ischemic heart conditions were observed by preserving LV function. • Efficacy diminished during re-perfusion.
**Statins and FTIs**	• Farnesyl pyrophosphate synthase was inhibited, beneficial for regulating RAS pathways.
**ACE inhibitors**	• Telmisartan downregulated RAS-ERK pathway effects and enhanced PI3K-Akt-eNOS signaling. • Reduced myocyte size, fibrosis, and AF incidence.
**Other treatments**	• Carabin: It regulated calcineurin and Ras signaling. • Raf kinase inhibitor protein: It enhanced β-adrenergic pathway activation. • Cyclosporine: It inhibited calcineurin, mitigating cardiac hypertrophy. • Rapamycin: It inhibited JNK and Akt/mTOR, reversing hypertrophy in genetic models.

**Note:** [3, 12, 14-16, 18, 19, 39-41].

## LIST OF ABBREVIATIONS

ATP: Adenosine Triphosphate
AgII: Angiotensin II
AF: Atrial Fibrillation
ASD: Atrial Septal Defects
ADHD: Attention Deficit Hyperactivity Disorder
Ca^2+^: Calcium
CFC: Cardio-Facio-Cutaneous
CDC 42: Cell Division Control Protein 42
CRISPR: Clustered Regularly Interspaced Short Palindromic Repeats
CHD: Congenital Heart Disease
CS: Costello Syndrome
Cas9: CRISPR-associated Protein 9
cAMP: Cyclic Adenosine Monophosphate
DCM: Dilated Cardiomyopathy
eNOS: Endothelial Nitric Oxide Synthase
ERK: Extracellular Signal Regulated Kinase
FTIs: Farnesyl Transferase Inhibitors
FOXO: Forkhead Box Transcription Factors of Class O
GPCRs: G-protein Coupled Receptors
GATA4: GATA Binding Protein-4
GSK: Glycogen Synthase Kinase
GAB: GRB2-associated Binding Protein
GRB2: Growth Factor Receptor-Bound 2
GAP: GTPase-activating Protein
GEF: Guanine Nucleotide Exchange Factor
GRP1: Guanine Nucleotide-releasing Protein1
GDP: Guanosine Diphosphate
GTP: Guanosine Triphosphate
H-RAS: Harvey Rat Sarcoma Virus
HF: Heart Failure
HCM: Hypertrophic Cardiomyopathy
JNK: Jun NH2-terminal Kinase
JMML: Juvenile Myelomonocytic Leukemia
K-RAS: Kirsten Rat Sarcoma Virus
LTCC: L‐type Calcium Channel
LV: Left Ventricular
LS: Legius Syndrome
LZTR1: Leucine Zipper-like Transcription Regulator 1
mTOR: Mammalian Target of Rapamycin
MAPK: Mitogen-activated Protein Kinase
MEK: Mitogen-activated Protein Kinase Kinase
MEF2: Myocyte Enhancer Factor-2
N-RAS: Neuroblastoma Rat Sarcoma Virus
NF1: Neurofibromatosis Type 1
NS: Noonan Syndrome
NE: Norepinephrine
NSML: NS with Multiple Lentigines
NF-κB: Nuclear Factor Kappa B
NFAT‐3: Nuclear Factor of Activated T Cells
PI3K: Phosphoinositide 3‐Kinase
PLN: Phospholamban
PLC: Phospholipase C
PKA: Protein Kinase A
Akt: Protein Kinase B
PVS: Pulmonary Valve Stenosis
RAF: Rapidly Accelerated Fibrosarcoma
R-RAS: RAS-related
Rac1: Ras-related C3 Botulinum Toxin Substrate 1
RAS: Rat Sarcoma
RTK: Receptor Tyrosine Kinase
RMS: Rhabdomyosarcoma
RyR: Ryanodine Receptor
SERCA: Sarco-Endoplasmic Reticulum Ca++ ATP‐ase
SOS: Son of Sevenless
SPRED1: Sprouty-related EVH1 Domain Containing 1 Src
SH2: Src Homology 2
Shp2: domain-containing Protein Tyrosine Phosphatase
SAPK: Stress-activated Protein Kinase
SCD: Sudden Cardiac Death
PTPN11: Tyrosine Protein Phosphatase Non-Receptor Type 11
Val: Valine
